# The Best of Medical Humour

**Published:** 1991-09

**Authors:** M. G. Wilson


M. G. Wilson

**THE BEST OF MEDICAL HUMOUR**
A collection of articles, essays, poetry, and letters published in the medical literature.
Compiled and edited by **Howard J. Bennett**
Hanley and Belfuss. Philadelphia $30

My interest in this book was aroused when I discovered that it contained an article 'Prolonged haemorrhage following nail clipping' by John Burton whose inspired writings frequently appear in this journal. His article was found under the section 'Case Reports' and on reading further discovered that the patient was a 2 year old male budgerigar employed as an entertainer at a local primary school and that it was reprinted with permission from the Lancet. That was a good start. Of course humour is a serious business and the book is divided into 14 sections under various headings such as 'Medical School', Internship and Residency', 'Medical Language', 'Poetry', 'Case Reports' etc. My most serious objection to the book is that there is no index and it took me a long time to locate Dr. Burton's article. However the subject classification will be of practical use. Those about to make an after dinner speech may find that it helps them find inspiration. In fact most of the selections are of a high order and one can browse through the book and even find it difficult to put down. The fact is that most of us seldom find occasion to take up such a book. It is a pity, this book is very worthwhile taking up and moreover, definitely something to give to your medical husband/wife/houseman/registrar for Christmas/birthday — which decides me what next to give my daughter-in-law and mark you, I shall shell out the necessary. I shall keep my reviewer's copy.

